# American Medical Association

**Published:** 1860-01

**Authors:** 


AMERICAN MEDICAL ASSOCIATION.—We copy the subjoined resolutions, with the accompanying comments, from the November number of the *Cincinnati Lancet and Observer*, edited by Dr. Stephens and Dr. Murphy. They meet with our most hearty commendation. The sentiments which they breathe, and which are, in every respect, so just and proper, we have long contended for in the pages of the Review:—

"We have received the following resolutions, which were presented to the last meeting of the Illinois State Medical Society, and laid upon the table until its next annual session. We insert them with pleasure, and with but brief comment. It is certainly unfortunate that precedent has so far established the custom of selecting the President of our American Medical Association from the locality of its annual meeting; and this custom has been freely and fully commented upon by a large proportion of the medical press of the country—so freely, indeed, that we scarcely feel called upon, in this connection, to enter into any particular discussion of the question. It would seem that, when the annual meeting of the National Association convenes, the courtesy of the members becomes more enlarged than when in State conventions or sitting under editors' tables; and thus far the prevailing disposition has been to yield the presidency to the claims of the city whose hospitality is being enjoyed. Fortunately, thus far the gentlemen who have been elevated to that honorable position have not been such as to bring any discredit to the position of the association—a fact, however, which does not affect the argument.

"'*Whereas*, The American Medical Association is a national organization, composed of delegates and members from all parts of the United States, meeting on terms of perfect equality; therefore—

"'*Resolved*, That, in the opinion of this society, all the officers of the association should be selected strictly with reference to merit, and without any regard to their places of residence.

"'*Resolved*, That the custom of selecting the president of the association exclusively from the profession of the city in which the annual meeting is held, is not only derogatory to the general character of the organization, and calculated greatly to lessen the honor which should attach to that office, but past experience has shown that it leads directly to local divisions, jealousies, and injurious, partisan strife.

"'*Resolved*, That the delegates from this society to the association be, and are hereby instructed to use their influence to abrogate the custom alluded to in the preceding resolution.

"'*Resolved*, That the Secretary be directed to furnish copies of the foregoing resolutions to other State and local medical societies, and ask their attention to the same.'"

